# Optimization of the ZigBee Routing Algorithm for the Beidou Sugar Beet Environmental Monitoring System

**DOI:** 10.3390/s26113414

**Published:** 2026-05-28

**Authors:** Hongbo Yu, Yu Liu, Jiadi Wei

**Affiliations:** School of Communication and Electronic Engineering, Qiqihar University, Qiqihar 161006, China; 2025936295@qqhru.edu.cn (Y.L.); 2023935735@qqhru.edu.cn (J.W.)

**Keywords:** Beidou system, sugar beet environmental monitoring, ZigBee networks, energy-balanced routing, wireless sensor networks

## Abstract

In remote areas where sugar beets are grown on a large scale, inadequate ground-based communication networks can easily lead to information silos in farmland, as well as technical challenges such as uneven node power consumption and short lifespans during the long-term operation of wireless sensor networks. To address these challenges, a real-time field environment monitoring system for sugar beet fields based on the Beidou satellite system and ZigBee wireless sensor networks has been developed, employing a three-tier architecture comprising a perception layer, a network layer, and an application layer. The system uses ARM as the core of the data acquisition nodes and integrates sensors for temperature, humidity, light intensity, atmospheric pressure, and dissolved oxygen with a Beidou positioning module. Field data are aggregated via a ZigBee mesh network and transmitted remotely using a dual-link Beidou short message protocol. To prevent uneven energy consumption in ZigBee networks, an improved energy-balanced routing algorithm, Energy-Balanced Low-Energy Adaptive Clustering Hierarchy (EB-LEACH), is proposed. By optimizing cluster head election, adaptive competition radius mechanisms, and inter-cluster multi-hop routing strategies through multi-factor weighting, the algorithm achieves a globally balanced distribution of network energy consumption. Our experimental tests demonstrate that, compared to the traditional LEACH protocol, this algorithm increases the number of rounds until the first node fails by 87.3%, extends the network half-life by 110.48%, and improves total packet delivery by 118.3%. Our test results indicate that the improved routing algorithm performs better, and the accuracy of the sensor measurements meets the practical requirements for environmental monitoring in sugar beet fields.

## 1. Introduction

Sugar beets are one of the most important sugar crops in northern China, and their yield and quality are critical to the security of the national sugar supply. Since Heilongjiang Province is a major sugar beet production area, accurately monitoring field environmental parameters in the region is essential due to its large-scale cultivation. Sugar beets primarily grow in environments with temperatures ranging from 15 °C to 25 °C, relative humidities between 55% and 75%, light intensities of 20,000 to 50,000 lux, oxygen levels of 4 mg/L to 12 mg/L, and atmospheric pressures between 950 hPa and 1050 hPa. However, large-scale sugar beet cultivation areas are generally located in remote regions with sparse populations and poor infrastructure, making it difficult for traditional monitoring systems to ensure stable remote data transmission, resulting in widespread “information silos.” At the same time, wireless sensor networks deployed across large-scale farmlands face technical issues during long-term operation, including uneven node energy consumption, prominent energy holes, and short network lifespans. These environments pose unique challenges for the deployment of environmental monitoring systems: First, ground-based communication networks currently have limited coverage in the main sugar beet-producing regions, with many farmlands located in 4G and 5G signal dead zones, preventing environmental data collected in the fields from being transmitted promptly to remote monitoring centers, thus creating information silos. Second, field monitoring nodes are far from the power grid and must rely on battery or solar power. Ensuring the long-term, stable operation of the system under limited energy supply is a major factor limiting the practical application of monitoring systems. Furthermore, when the monitoring area is large, precise coordinates are required to pinpoint specific locations, placing higher demands on positioning technology.

The application of China’s Beidou Satellite Navigation System offers a completely new technical approach to addressing these issues. The Beidou system has two primary functions: high-precision positioning and short message communication [1,2]. Short message communication distinguishes the Beidou system from other global satellite navigation systems such as GPS and GLONASS [3], enabling users to transmit two-way short messages via satellites [4,5,6]. Applying Beidou short message communication to sugar beet environmental monitoring allows for reliable data transmission in remote areas without ground network coverage, effectively resolving the issue of information silos. Additionally, a single terminal can simultaneously obtain location information and conduct data communication, achieving an integrated design for positioning and communication, which simplifies the system architecture and reduces hardware costs. Compared to pseudo-satellite systems, the combination of ZigBee and Beidou is better suited for outdoor farmland scenarios. BeiDou provides positioning services and, when combined with ZigBee’s low-power networking capabilities, enables cost-effective farmland positioning and environmental monitoring. Pseudo-satellite systems are expensive to deploy and are better suited for high-precision positioning in urban areas with dense coverage gaps rather than large-scale open farmland areas [7].

In large-scale sugar beet field cultivation scenarios, ZigBee technology offers advantages over mainstream long-range ground communication technologies such as LoRa and NB-IoT. LoRa requires dense gateway deployment and has limited coverage, while NB-IoT relies on cellular base station infrastructure, resulting in poor signal coverage in remote agricultural areas. In contrast, ZigBee has a lower latency for short-range, high-frequency communication and offers better real-time data transmission than LoRa. Although LoRa offers long transmission distances and strong penetration, its low data rates and high latency make it unsuitable for high-frequency data collection in the field. At the same time, ZigBee features low hardware costs and controllable power consumption, meeting the requirements for low-cost networking in remote sugar beet growing areas. Because of this, ZigBee has become the mainstream choice for wireless sensor networks in field-level local area networks [8,9,10]. A typical ZigBee network can accommodate hundreds of nodes, providing coverage across entire cultivation areas spanning tens of square kilometers. However, the greatest challenge facing the large-scale deployment of ZigBee networks is energy consumption. Cluster head nodes in the network must handle both forwarding and data fusion tasks, resulting in significantly higher energy consumption compared to ordinary nodes. If cluster head election is improperly managed, some nodes may deplete their energy prematurely, creating an energy gap that shortens the lifespan of the entire network [11]. Although the classic LEACH protocol employs a random rotation mechanism to balance energy consumption, it performs poorly in long-term monitoring environments like large-scale sugar beet fields due to its inherent randomness—which leads to uneven cluster head distribution and fails to account for issues such as remaining node energy.

## 2. System Grid Overall Design Scheme

**Figure 1:**
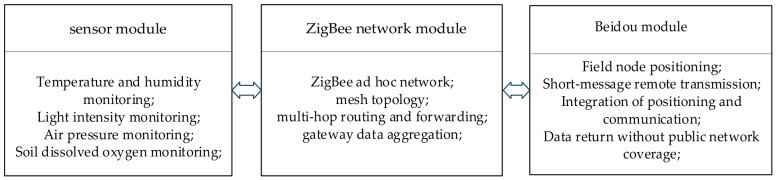
System functional framework diagram.

The overall framework of this system primarily consists of the Beidou satellite system, a sensor network, and a communication network module. As shown in Figure 1, the system employs a three-layer network architecture: The perception layer comprises data collection nodes deployed in sugar beet fields, which integrate sensors for temperature, humidity, light intensity, atmospheric pressure, dissolved oxygen, and other parameters, as well as Beidou positioning modules, and form a self-organizing network via a ZigBee mesh network. The network layer centers on gateway aggregation nodes, which are responsible for receiving and fusing field data uploaded by the various data collection nodes. The transport layer uses the Beidou short message service as the primary link and 5G as the backup link to remotely transmit the processed data back to the remote monitoring center at the application layer.

**Figure 2:**
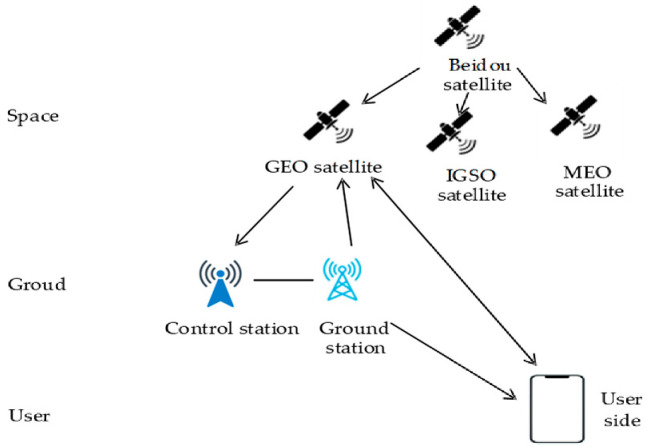
Beidou network system.

The design functions of the Beidou module are shown in Figure 2 and include the following: (1) determining the latitude and longitude coordinates of each data collection node within the monitoring area, packaging the environmental data, and transmitting the data to the Beidou satellites via the short message service (SMS); (2) the satellites forwarding the data packets to the ground receiving station; (3) the ground receiving station comparing and verifying the received data against the transmitted data to confirm the integrity of the communication [12,13,14].

The key design features of the communication network module are as follows: Each data collection node gathers environmental data and transmits them in the form of data packets to the ZigBee coordinator node. Upon receiving the data packets, the coordinator node aggregates the information and sends it via the Beidou satellite communication system to the Beidou terminal module. From there, the data are transmitted to the monitoring center via a ground receiving station, enabling managers to receive real-time field environmental data.

## 3. System Hardware Design

### 3.1. System Overall Design Scheme

**Figure 3:**
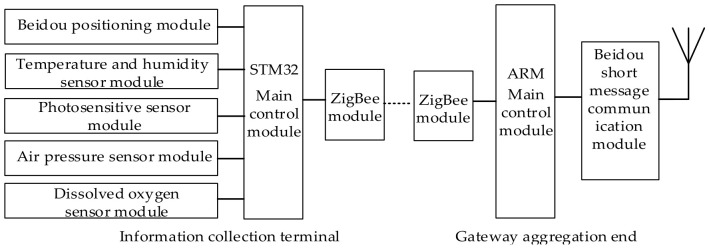
System architecture diagram.

The data acquisition end of this system primarily consists of the following components: the STM32 main control module, ZigBee end nodes, a Beidou positioning module, and various sensor modules. The gateway aggregation node end includes key modules such as the ZigBee gateway aggregation node, the ARM main control module, and the Beidou positioning module, as shown in Figure 3. The control module of the data acquisition node uses the STM32F103ZET6 microcontroller (STMicroelectronics, Geneva, Switzerland). It is primarily responsible for initializing and controlling the Beidou positioning module and various sensor modules, receiving and processing data collected from sensors (temperature, humidity, light intensity, air pressure, and dissolved oxygen), and managing communication between the ZigBee module and the gateway node. The gateway aggregation node utilizes an ARM-based i.MX6ULL Pro core board and is responsible for receiving field data aggregated from the ZigBee network, transmitting the processed information to the remote monitoring center via the Beidou short message module (primary link), and displaying data and status information on a local LCD screen.

Within our intelligent agricultural monitoring framework, the Beidou system is not merely a standalone positioning and communication auxiliary module, but is rather an indispensable core component that is deeply integrated with the EB-LEACH routing optimization mechanism. Beidou provides high-precision geographic coordinates to distributed sensor nodes, enabling the EB-LEACH protocol to perform network partitioning and optimize cluster head deployment and transmission distances, thereby effectively balancing network energy consumption and reducing long-distance transmission overhead. At the same time, BeiDou’s short message communication capability enables the transmission of remote monitoring data from vast, remote agricultural areas. Since the BeiDou system achieves seamless, comprehensive coverage without relying on ground-based communication infrastructure, it is particularly well suited for large-scale, decentralized, and unmanned agricultural monitoring scenarios. Furthermore, Beidou’s high-precision timing capabilities assist EB-LEACH in optimizing time-division multiple access (TDMA) slot scheduling and synchronized data acquisition, thereby reducing the probability of data collisions and minimizing additional energy consumption. Consequently, compared to traditional monitoring architectures based on low-power wide-area networks (LPWAN), the integrated framework incorporating the Beidou system offers distinct advantages in terms of positioning support, routing optimization, coverage stability, and deployment costs.

### 3.2. ZigBee Module Interface Circuit Design

**Figure 4:**
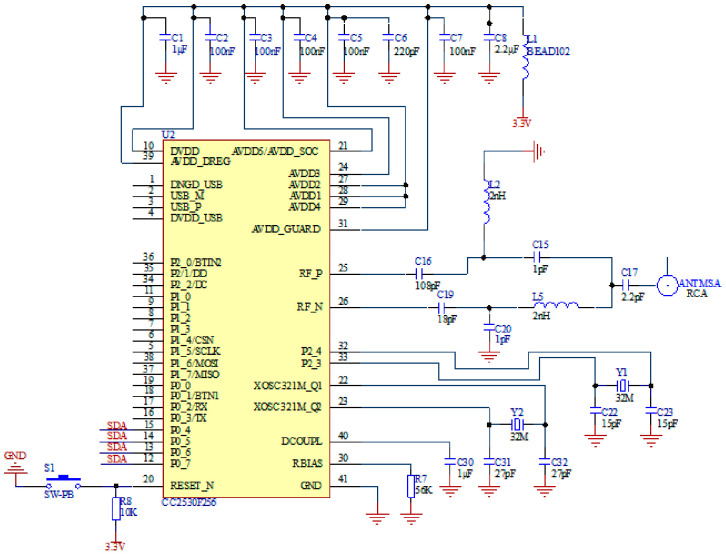
ZigBee module interface circuit diagram.

The ZigBee communication module uses the CC2530 chip, which integrates an 8051 microcontroller core and a 2.4 GHz wireless transceiver compliant with the IEEE 802.15.4 standard [15,16,17]. The CC2530 connects to the STM32 host chip via a UART interface, with communication parameters configured as a baud rate of 115,200, 8 data bits, 1 stop bit, and no parity. The RF output pin is converted to a single-ended 50 Ω impedance via an LC matching network, and the antenna area is kept clear, as shown in Figure 4.

As the core of the wireless communication system, the CC2530 module first completes initialization and establishes a ZigBee ad hoc network upon power-up. It then receives data collected by various sensors and location information from the Beidou positioning module in real time and finally transmits data frames to the network coordinator node via the ZigBee ad hoc network. This entire process integrates Beidou signal acquisition, location resolution, and wireless data transmission into a single solution.

### 3.3. Design of the Beidou Module Interface Circuit

**Figure 5:**
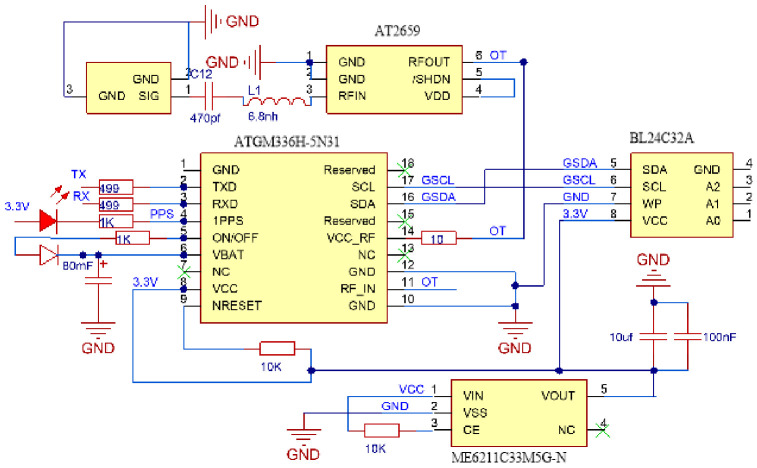
Beidou module interface circuit diagram.

The Beidou system utilizes the ATGM336H-5N31 high-performance BDS/GNSS positioning module, which supports multi-system simultaneous positioning using Beidou, GPS, and other systems. The module outputs positioning data in standard NMEA-0183 format via a UART interface at a baud rate of 9600 bps. The module is powered by a single 3.3 V supply provided by the ME6211C33M5G-N LDO regulator (Nanjing Micro One Electronics Co., Ltd., Nanjing, China) and is equipped with an AT2659 low-noise amplifier (Hangzhou Icofchina Microelectronics Co., Ltd., Hangzhou, China) to enhance reception sensitivity, as shown in Figure 5.

This Beidou positioning module integrates a complete signal processing workflow that includes signal acquisition, tracking, navigation message decoding, and positioning calculation. Its built-in antenna can receive RF navigation signals in agricultural environments, acquire visible satellites, and suppress environmental interference such as crop shading and terrain undulations. Once signal acquisition is complete, the carrier loop and code loop continuously track satellite pseudorange and carrier phase to maintain stable signal synchronization. The demodulated navigation message contains ephemeris data, satellite clock parameters, and orbital information. The STM32 microcontroller calculates satellite coordinates based on the decoded ephemeris and, using the pseudorange observation equation and least-squares algorithm, accurately determines the node’s latitude and longitude. Finally, positioning information—including longitude, latitude, altitude, and the number of satellites—is output via the standard NMEA-0183 protocol and transmitted to the ZigBee module to enable multi-source data fusion for agricultural applications. The circuit diagram of the Beidou module is shown in Figure 5. After the module is powered on, it begins receiving satellite signals. In an open outdoor environment, the positioning process can be completed within one minute, and location information is output once per second. To parse Beidou data, the $GNRMC message provided by the Beidou positioning core must be used. The format of this message is as follows:

$GNRMC, UTC time, status, latitude, n/s, longitude, e/w, speed, heading,

date, magnetic declination, checksum

When the status in the message is set to “A”, positioning is successful. Overall, the Beidou positioning process includes (1) enabling serial port reception and reading NMEA data byte by byte; (2) detecting the $GNRMC frame header to filter valid messages; (3) splitting the message fields by commas; (4) determining whether the positioning status is set to “A”; (5) extracting latitude and longitude in degree–minute format; (6) converting to decimal latitude and longitude coordinates; and (7) outputting the positioning result to complete the position acquisition. The message obtained in this experiment is as follows:

$GNRMC,082515.000, A,4721.1568, N,12356.7845, E,0.00,123.4,200326, A*4B

The message parsing results are as follows: UTC time 20 March 2026 08:25:15; positioning status valid; converted coordinates: 47.352613° N, 123.946408° E. This corresponds to Qiqihar City, Heilongjiang Province, indicating that the BeiDou positioning system is capable of accurately acquiring and parsing node location information.

### 3.4. Interface Circuit Design for the Temperature and Humidity Sensing Module

**Figure 6:**
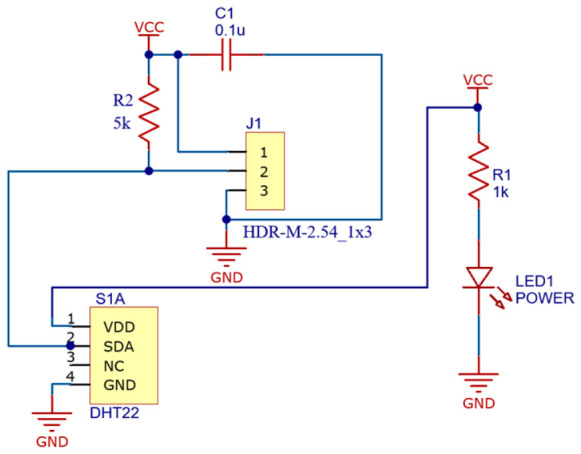
Temperature and humidity sensor interface circuit diagram.

The temperature and humidity sensing module uses the AM2302 digital temperature and humidity sensor (Guangzhou Aosong Electronics Co., Ltd., Guangzhou, China), which is an upgraded version of the DHT11 and offers higher measurement accuracy and a wider measurement range. The sensor integrates a capacitive humidity sensing element and an NTC temperature sensing element. It communicates with the STM32 microcontroller via a single-bus protocol and outputs calibrated digital signals. The interface circuit design is shown in Figure 6.

Upon receiving a startup signal from the host, the AM2302 sensor module acquires analog environmental temperature and humidity data, converts the data into digital signals, and transmits 40-bit data serially via a single-wire bus. The main control unit receives and verifies the data, parses them to obtain the actual temperature and humidity values, and uploads them to the coordinator node via a ZigBee wireless network.

### 3.5. Interface Circuit Design for the Photosensitive Sensor Module

**Figure 7:**
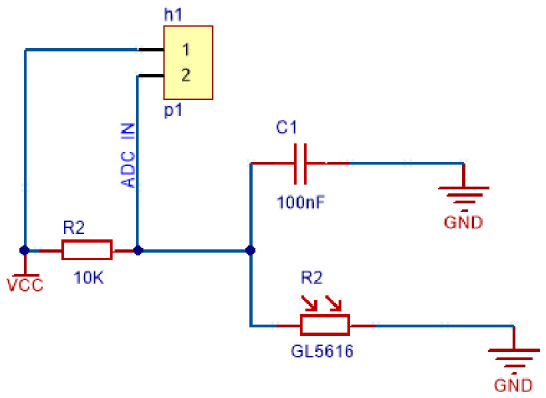
Schematic diagram of the photosensitive sensor interface circuit.

The photosensitive sensor uses a GL5616 photoresistor (Shenzhen Jing Chuang He Li Technology Co., Ltd., Shenzhen, China), with a resistance range of 5–10 kΩ (10 lux) in light and ≥1 MΩ in darkness, and a response time of ≤30 ms. The GL5616 photoresistor is paired with a simple voltage divider circuit. The voltage at the divider tap is acquired via the STM32′s built-in ADC module and converted into an illuminance value through software. The interface circuit design is shown in Figure 7.

Finally, after multi-sensor data fusion processing, the data are uploaded to the coordinator node via the ZigBee network. This solution features a simple circuit design and low cost, meeting the requirements for continuous illuminance measurement in beet field environmental monitoring.

### 3.6. Circuit Design of the Barometric Pressure Monitoring Module

**Figure 8:**
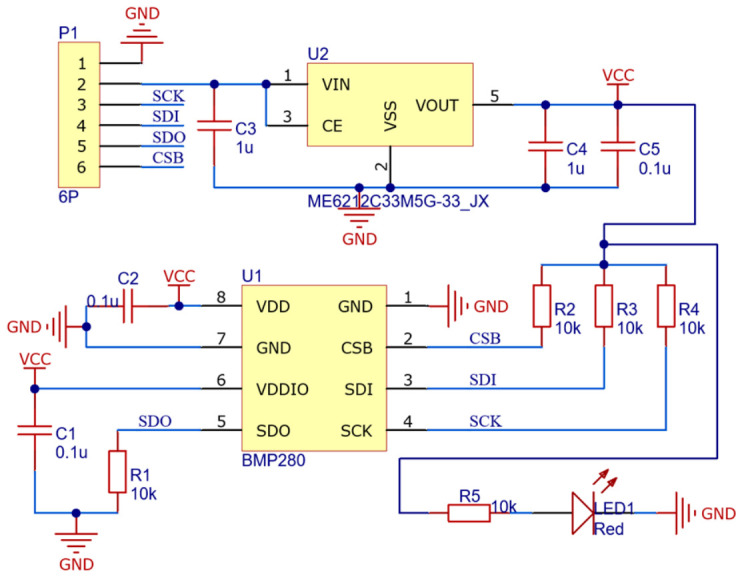
Barometric pressure sensor interface circuit diagram.

The BMP280 digital barometric pressure sensor is used for pressure measurement. This high-precision sensor has a measurement range of 300 hPa to 1100 hPa, with an accuracy of ±1.0 hPa. It features built-in temperature compensation and supports I^2^C and SPI interfaces, enabling simultaneous output of atmospheric pressure and temperature data. This design uses the I^2^C interface to connect to the STM32 microcontroller, resulting in a simple and reliable circuit. The interface circuit design is shown in Figure 8.

The workflow begins with the STM32 initializing the GPIO and I^2^C buses after power-up. It then verifies that communication with the BMP280 is functioning properly by reading the device ID and configures the sensor’s operating mode and sampling parameters. Next, the STM32 reads the factory calibration data, triggers barometric pressure acquisition, retrieves the raw digital values via I^2^C, and performs temperature and pressure compensation calculations using the calibration parameters to obtain the actual ambient barometric pressure value.

### 3.7. Dissolved Oxygen Sensor Interface Circuit

**Figure 9:**
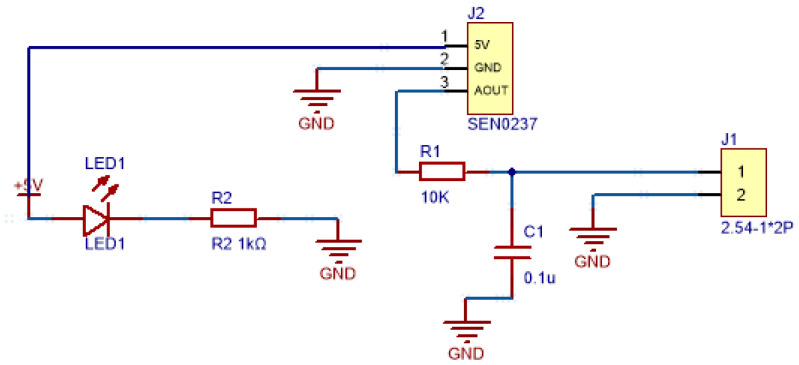
Dissolved oxygen sensor interface circuit diagram.

The interface circuit design is shown in Figure 9. The dissolved oxygen monitoring system uses the SEN0237 dissolved oxygen sensor (DFRobot, Shanghai, China), which outputs an analog voltage signal ranging from 0 to 3.0 V, corresponding to a dissolved oxygen concentration of 0 to 20 mg/L. The sensor requires a temperature sensor for temperature compensation.

The voltage signal is acquired via the STM32’s built-in ADC module and converted into a dissolved oxygen concentration value through software. The STM32 initializes the SEN0237 sensor via I2C, collects raw data on dissolved oxygen and temperature, and, after compensation and filtering, outputs stable water quality monitoring parameters that are uploaded to the ZigBee network.

## 4. System Software Design

The system software consists of three components: the data acquisition node program, the gateway aggregation node program, and the remote monitoring center program. Upon power-up, each node first enters an initialization state to perform hardware self-tests, clock configuration, and peripheral initialization. The data acquisition node periodically collects environmental data from the sugar beet field via sensor modules, including parameters such as temperature, humidity, light intensity, atmospheric pressure, and dissolved oxygen. Simultaneously, it obtains the node’s location information through the Beidou module, then transmits the collected data to the ZigBee coordinator node according to a routing algorithm designed as follows.

### 4.1. Design of the EB-LEACH Routing Algorithm

To address the shortcomings of the traditional LEACH algorithm, we have designed an improved energy-balanced routing EB-LEACH algorithm, one which presents clear, differentiated characteristics and inherent innovations. DE-LEACH adopts a joint weighting mechanism of node coordinate distance and residual energy that relies on additional positioning information and brings extra computational and communication overhead. In contrast [18], EB-LEACH reduces the frequency and complexity of node location and distance calculation and uses distance parameters only for lightweight auxiliary judgment in cluster head election and competition radius adjustment. Unlike EE-LEACH, which employs a centralized scheduling strategy and always preferentially selects nodes with the maximum residual energy [19], leading to the premature failure of high-energy nodes, EB-LEACH adopts a dynamic average energy threshold to realize fair rotation of eligible nodes and avoid overconsumption of individual high-performance nodes. In addition, most conventional energy-balanced clustering protocols adopt a fixed energy threshold, which cannot adapt to the continuous attenuation of network energy, whereas EB-LEACH implements an adaptive threshold update mechanism that dynamically adjusts the screening criterion following the overall energy decline of the network. Consequently, EB-LEACH is not a simple combination of existing strategies, but a minimalist dynamic energy screening clustering mechanism with outstanding advantages in reducing computational complexity, controlling network overhead, and adapting to low-cost resource-limited sensor nodes.

The algorithm introduces improvements in two key areas: the cluster head election mechanism and network topology optimization. These enhancements are designed to achieve a more balanced distribution of network power consumption, thereby extending the network’s lifespan.

#### 4.1.1. An Improved Multi-Factor Weighted Cluster Head Election Algorithm

During the cluster head election phase, the EB-LEACH algorithm comprehensively considers a node’s remaining energy, geographical location, and network load conditions [20]. It represents a significant improvement to the threshold calculation formula of the traditional LEACH algorithm, with a new threshold calculation formula as follows:

(1) $$T{\left(n\right)}_{\mathrm{new}}=\left\{\begin{array}{l} \frac{p}{1-p(r\mathrm{mod}1/p)}\times [\alpha \times \frac{E_{\mathrm{current}}}{E_{\max}}+(1-\alpha )\times (1-\frac{d_{\mathrm{to}\_\mathrm{sink}}}{d_{\max}})]\;\;\;\;\;\;\;\;\;\;\mathrm{if}\;\;\;\;n\in G\\ \;\;\;\;\;\;\;\;\;\;\;\;\;\;\;\;\;\;\;\;\;\;\;\;\;\;\;\;\;\;\;\;\;\;\;\;\;\;\;\;\;\;\;\;\;\;\;\;\;\;\;\;\;\;\;\;\;0\;\;\;\;\;\;\;\;\;\;\;\;\;\;\;\;\;\;\;\;\;\;\;\;\;\;\;\;\;\;\;\;\;\;\;\;\;\;\;\;\;\;\;\;\;\;\;\;\;\;\;\;\;\;\;\mathrm{otherwise} \end{array}\right.$$

In this formula, P indicates the expected percentage of cluster heads relative to the total number of nodes, r this is the current round, $E_{current}$ indicates the node’s current remaining energy, $E_{max}$ is the initial energy of the node, $d_{to\_sink}$ is the Euclidean distance from the node to the aggregation node, $d_{max}$ is the maximum distance from any node in the network to the aggregation node, and α is the weighting factor (0<α<1), which is used to balance the importance of the energy factor and the distance factor in cluster head election [21,22,23]. This improvement ensures that nodes with higher residual energy, closer proximity to the convergence node, and lighter historical load have a higher probability of becoming the cluster head, thereby optimizing the quality of cluster head election across multiple dimensions.

The innovation of this improvement lies in the introduction of an energy factor $\alpha \times \frac{E_{\mathrm{current}}}{E_{\max}}$. Ensuring that nodes with higher remaining energy have a greater probability of becoming cluster heads prevents low-energy nodes from dying prematurely. The distance factor is calculated as follows:

(2) $$\left(1-\alpha )\times (1-\frac{d_{\mathrm{to}\_\mathrm{sink}}}{d_{\max}}\right)$$

This allows nodes closer to the convergence node to have a higher probability of being elected, thereby effectively reducing the energy consumption associated with long-distance communication.

#### 4.1.2. Adaptive Cluster Head Competition Mechanism

The EB-LEACH algorithm introduces a dynamic cluster head competition mechanism designed to optimize the spatial distribution of cluster heads within the network. It dynamically adjusts the competition radius of cluster heads based on the distance from a node to the nearest cluster head using the following formula:

(3) $$R_{\mathrm{comp}}=R_{0}\times (1-\beta \times \frac{d_{\mathrm{to}\_\mathrm{sink}}}{d_{\max}})$$

In this formula, $R_{0}$ is the basic competitive radius, and β is the distance adjustment factor (0<β<1). The design philosophy behind this mechanism is that nodes closer to the aggregation node have a larger competition radius and can assume more cluster head responsibilities, thereby balancing the network’s overall energy consumption. Nodes farther from the aggregation node have a smaller competition radius, which prevents them from consuming excessive energy when acting as cluster heads. This design effectively reduces the average number of hops required to transmit data to the aggregation node, thereby further optimizing energy consumption across the entire network.

In summary, the EB-LEACH algorithm establishes a dual adaptive optimization mechanism comprising adaptive energy screening criteria and adaptive competition radii. The algorithm first uses the real-time average energy across the entire network as the adaptive screening criterion, dynamically updating the selection threshold to eliminate nodes with low energy and ensure the overall quality of cluster head nodes. Building on this foundation, the algorithm sets a variable competition radius based on differences in node distances, granting nodes farther from aggregation nodes a larger coverage area. This forms larger cluster structures, reduces the number of hops for data forwarding at the network edge, and alleviates the high energy consumption issue faced by edge nodes. The dual adaptive mechanism collaboratively optimizes the network topology, balances energy consumption across the entire network, and significantly improves the stability and service life of the farmland monitoring network.

#### 4.1.3. Multi-Hop Routing

During the stable data transmission phase, the EB-LEACH algorithm uses multi-hop routing to transmit data from cluster heads to aggregation nodes, rather than having cluster heads communicate directly with base stations as in traditional LEACH. Cluster head nodes establish routing tables to aggregation nodes based on received signal strength and select the path with the lowest energy consumption for data forwarding. For cluster heads located far from the aggregation node, multi-hop forwarding via nearby cluster heads effectively avoids the significant energy consumption associated with long-distance direct communication. At the same time, multi-hop transmission further balances the network load, preventing some cluster heads from dying prematurely due to an excessive forwarding workload.

#### 4.1.4. Algorithm Flow Design

**Figure 10:**
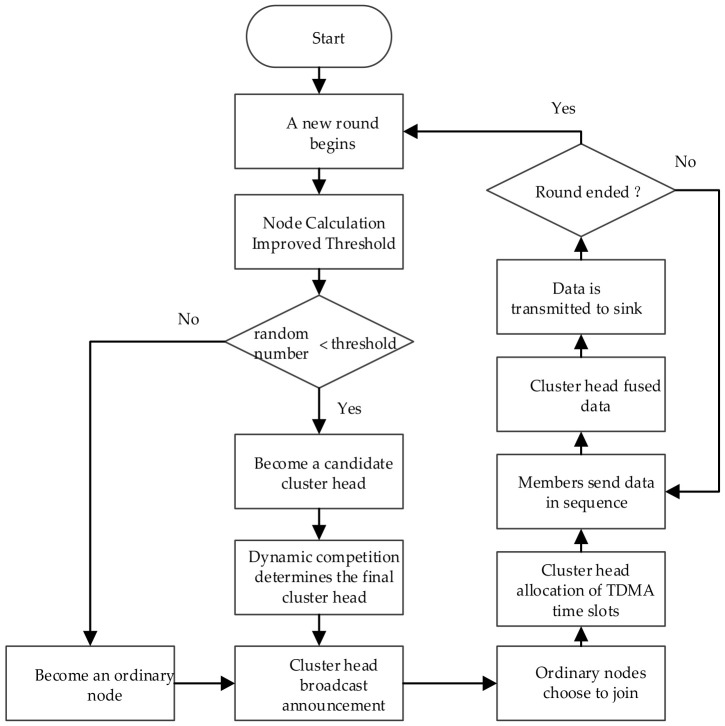
EB-LEACH algorithm workflow.

The EB-LEACH algorithm operates in cycles, with each cycle consisting of three core phases: cluster head election, cluster formation, and stable data transmission. The workflow is illustrated in Figure 10.

During the cluster head election phase, each node first determines whether it is eligible to participate in the election based on an improved threshold calculation formula. Specifically, a node generates a random number between 0 and 1; if this number is smaller than the calculated threshold, the node becomes a candidate cluster head. These candidate cluster heads then use a dynamic competition radius mechanism to determine which nodes will ultimately become cluster heads. During the cluster formation phase, the selected cluster head nodes broadcast an announcement message to the entire network.

Ordinary nodes select the most suitable cluster head based on the signal strength they receive, and, once selected, they send a join request to the corresponding cluster head. Upon receiving these requests, the cluster head assigns a dedicated TDMA timeslot to each joining member, thereby establishing a stable scheduling arrangement for internal communication. Once the stable data transmission phase begins, each member node within a cluster transmits the collected environmental data to its cluster head during its assigned time slot. The receiving cluster head node then fuses these data—primarily to reduce redundancy—and subsequently forwards the aggregated data step by step to the aggregation node via multi-hop routing.

This design is primarily intended to enable the network to adaptively respond to changes in the energy status of individual nodes. It is precisely this periodic mechanism of continuously re-electing cluster heads that ensures that the network structure can be dynamically optimized, allowing the EB-LEACH algorithm to automatically adjust the direction and focus of its selection strategy based on real-time changes in network energy distribution and topology. Of course, the ultimate goal is to achieve greater energy efficiency and significantly extend the service life of the entire system.

The EB-LEACH algorithm adopted in this paper addresses the issue of excessive energy consumption caused by frequent cluster head elections in the classic LEACH algorithm. This algorithm defines a 5-min interval as one complete data collection cycle and a 20-min interval as one complete data transmission cycle, with each data transmission round consolidating valid data from four consecutive 5-min collection periods. After the system completes 20 full data transmission cycles, it automatically performs a network-wide cluster head re-election and updates the clustering structure. This approach reduces the number of RF transmissions while ensuring the continuity of monitoring data, significantly lowering node energy consumption.

### 4.2. Simulation Testing of Routing Algorithms

Simulation verification of the EB-LEACH routing algorithm is performed using MATLAB R2020b, while the performance of the integrated Beidou and ZigBee monitoring platform is tested through field operations. To verify the effectiveness and superiority of the EB-LEACH algorithm, a simulation model of a sugar beet field monitoring network was developed using MATLAB. The simulation parameters, shown in Table 1, primarily included network topology, energy parameters, and algorithm parameters.

Simulation experiments were used to evaluate and analyze the algorithm’s performance in terms of network life cycle, energy efficiency, and data throughput. A 100-m-by-100-m two-dimensional simulation environment was created using MATLAB to simulate a sugar beet field environment.

**Figure 11:**
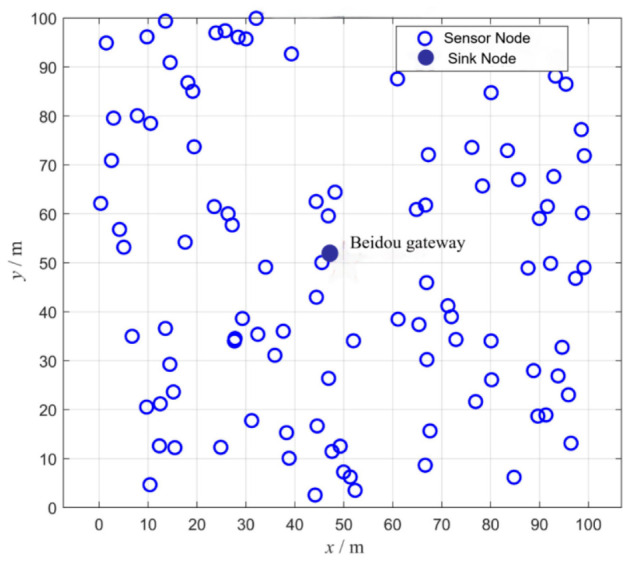
Network topology diagram.

We deployed 100 sensor nodes using a random uniform distribution, with the collection node fixed at the center of the area (50, 50). This network topology is shown in Figure 11. The collection node serves as the Beidou gateway and is responsible for aggregating various data from the ZigBee network.

To ensure simulation completeness and cover all sensor operating states, we have employed a standard first-order radio power consumption model. This model includes five types of power consumption: data acquisition, data transmission, data reception, data aggregation, and idle standby. All compared algorithms strictly use the same set of power consumption calculation formulas and physical parameters, ensuring that the simulation results are transparent, reproducible, and comparable.

(1) Energy consumption during data transmission

When a sensor node transmits data to a cluster head or base station, it automatically switches the channel loss model based on the transmission distance. Using the critical distance $d_{0}$ as the boundary, the free-space loss model is applied for short distances, while the multipath fading loss model is applied for long distances. The formula for calculating transmission power consumption is as follows:

(4) $$E_{\mathrm{Tx}}(I,d)=\left\{ \begin{array}{c} lE_{\mathrm{elec}}+l\varepsilon_{\mathrm{fS}}d^{2},d<d_{0}\\ lE_{\mathrm{elec}}+l\varepsilon_{\mathrm{mp}}d^{4},d\geq d_{0} \end{array}\right.$$

In the above equation, *l* is the bit length of the sample data; d is the transmission distance for nodes; $E_{elec}$ is the energy consumption coefficient for RF circuits;$\;\varepsilon_{fs}$ and $\varepsilon_{mp}$ denote the free-space and multipath fading gain factors, respectively; and $d_{0}=\sqrt{\varepsilon_{fs}/\varepsilon_{mp}}$ is the critical distance for the channel.

(2) Energy consumption for data reception

When a node receives a data packet, it consumes power only in the RF circuitry and does not consume power for signal amplification. $E_{\mathrm{Rx}\;}$, the energy consumption for a node, is calculated using the following formula:

(5) $$E_{Rx}(l)=lE_{elec}$$

(3) Data Aggregation Energy Consumption

The cluster head node must fuse and deduplicate data from multiple sources within the cluster to reduce the volume of redundant data transmitted. The energy consumption for data aggregation is expressed as follows:

(6) $$E_{\mathrm{Agg}}=lE_{da}$$

In this equation, $E_{\mathrm{Agg}}$ is the aggregate energy consumption data, and $E_{da}$ is the energy consumption coefficient per unit of data.

(4) Acquisition Power Consumption and Idle Power Consumption

To better reflect the operational characteristics of real-world agricultural sensors, this paper introduces node data acquisition power consumption and idle standby power consumption. Sensors generate acquisition Power Consumption when collecting environmental data such as temperature and humidity, whereas in practical sensing systems the acquisition energy is primarily determined by sensor operating current, acquisition duration, warm-up time, and analog front-end activity. Nodes that are not communicating remain in a low-power idle state, maintaining a baseline power consumption for monitoring. The specific formula is as follows:

(7) $$\left\{ \begin{array}{c} E_{\mathrm{Sense}}=I_{\mathrm{sen}}V_{\mathrm{dd}}t_{\mathrm{acq}}+E_{\mathrm{warm}}+E_{\mathrm{afe}}\\ E_{\mathrm{Idle}}=P_{\mathrm{Idle}}t \end{array}\right.$$

$E_{Sense}$ denotes the acquisition power consumption of a single node; $I_{sen}$ denotes the operating current of the sensor, $V_{dd}$ denotes the supply voltage, $t_{acq}$ denotes the duration of a single acquisition, $E_{warm}$ denotes the warm-up energy consumption of the sensor, $E_{afe}$ denotes the power consumption of the analog front-end circuit, $E_{Idle}$ is the idle power consumption of nodes, $P_{Idle}$ is the idle power consumption for the duration of the idle period, and t denotes the idle standby time.

(5) Total Network Energy Consumption

The total network energy consumption is calculated by summing the energy consumption of all sensor nodes in the network, providing a comprehensive reflection of the algorithm’s energy optimization performance. Its formula is as follows:

(8) $$E_{\mathrm{Total}}=\sum (E_{\mathrm{Sense}}+E_{\mathrm{Tx}}+E_{\mathrm{Rx}}+E_{\mathrm{Agg}}+E_{\mathrm{Idle}})$$

All simulation processes described in this paper are based on iterative calculations using the aforementioned energy consumption formula. Evaluation metrics such as the number of surviving nodes, total remaining energy across the network, and throughput are all derived from a unified energy consumption model, ensuring the fairness of the experiments and the reproducibility of the results.

#### 4.2.1. Network Life Cycle Analysis

**Figure 12:**
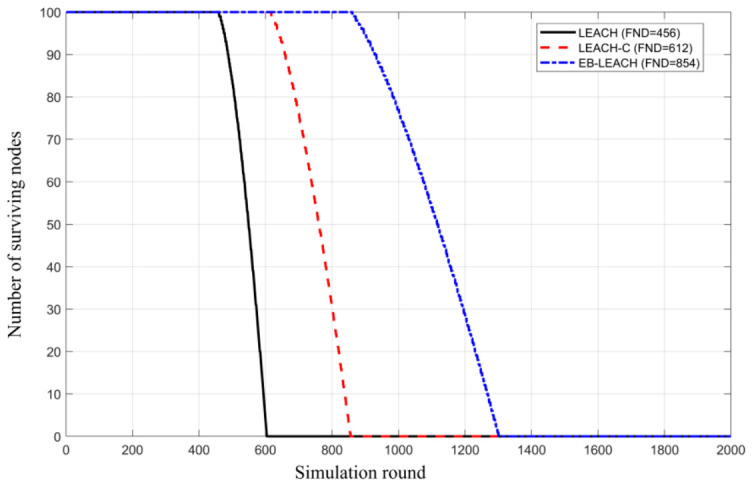
Comparison of network life cycles.

The network life cycle is the primary metric for evaluating the performance of routing algorithms in wireless sensor networks. MATLAB simulations were used to determine how the number of surviving nodes varies with the number of simulation rounds for the three algorithms, as shown in Figure 12. Our simulation results show that the EB-LEACH algorithm performs exceptionally well in extending the network’s lifespan. The first node failure in the traditional LEACH algorithm occurred at round 456, while the LEACH-C algorithm delayed this to round 612. The EB-LEACH algorithm significantly delayed the first node failure to round 854, representing an improvement of 87.3% and 39.5% over LEACH and LEACH-C, respectively.

In terms of network half-life (50% node failure), the EB-LEACH algorithm reached the 1105th round, whereas LEACH and LEACH-C reached only the 525th and 780th rounds, respectively. When the network completely fails, the EB-LEACH algorithm extends network survival time by approximately 30.3% compared to traditional LEACH. This significant performance improvement is primarily attributed to the energy- and distance-based cluster head election mechanism in the EB-LEACH algorithm, which effectively prevents the premature death of low-energy nodes and achieves balanced energy consumption across the entire network.

#### 4.2.2. Analysis of Network Energy Consumption Characteristics

**Figure 13:**
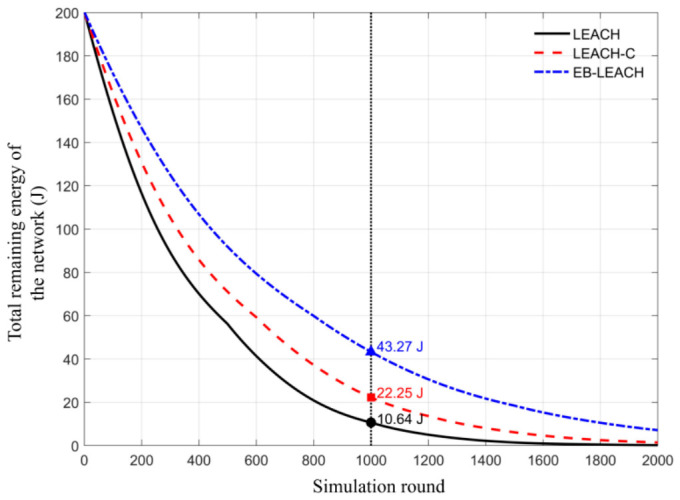
Curve of total residual energy in the network.

Since network energy consumption characteristics directly impact the system’s sustainability, simulation tests were conducted to compare these characteristics. Figure 13 illustrates the trend of total remaining network energy over the simulation rounds for the three algorithms.

In terms of the energy consumption rate, the traditional LEACH algorithm exhibited a rapid decline in energy over the first 500 rounds, with only 5.32% of the total network energy remaining by the 1000th round. The LEACH-C algorithm improved energy efficiency to some extent through centralized cluster head selection, maintaining 11.13% of the total network energy by the 1000th round. The EB-LEACH algorithm, however, demonstrated the best energy retention capability, with 21.64% of the total network energy remaining at the 1000th round. Its energy consumption rate was reduced by 17.23% and 11.82% compared to the LEACH and LEACH-C algorithms respectively.

A further analysis of the energy consumption balance revealed that the EB-LEACH algorithm exhibited the lowest variance in energy consumption across all nodes, indicating that its energy balancing mechanism effectively prevents the occurrence of energy black holes. This balanced energy consumption pattern enables the network to maintain useful coverage for longer periods, providing reliable assurance for the continuous monitoring of the beet growing environment.

#### 4.2.3. Data Throughput Performance Analysis

**Figure 14:**
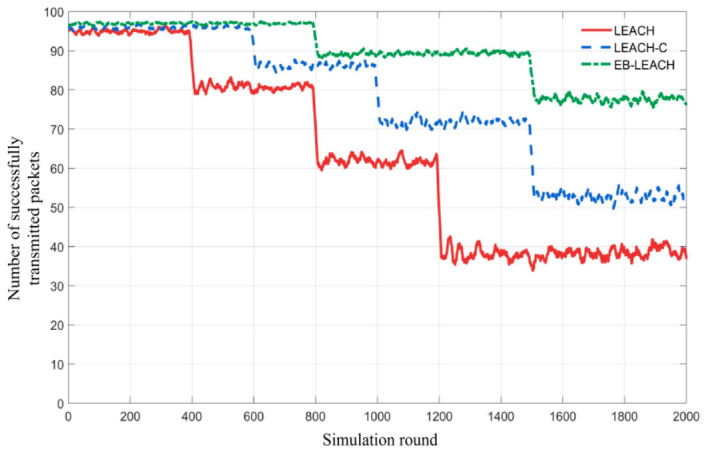
Comparison of successful packet transmission rates.

Data throughput reflects the monitoring system’s ability to effectively collect field environmental data. Figure 14 shows the number of data packets successfully transmitted to the aggregation node per round under the three algorithms.

As shown in Figure 14, the data throughput curves for all three algorithms exhibit a marked drop at specific rounds (approximately 400 rounds for LEACH, 800 rounds for LEACH-C, and 1200 rounds for EB-LEACH). Analysis suggests that these drops are closely related to the failure of the first critical node in the network. Taking the LEACH algorithm as an example, the first node fails due to energy depletion at approximately 400 rounds. This node is responsible for both collecting and forwarding data within its own cluster and acting as a communication relay for adjacent clusters. Its failure prevents data from being uploaded in that region, resulting in the first noticeable drop in throughput. As the number of failed nodes increases, the network gradually fragments into multiple isolated regions, leading to irrecoverable data loss and a step-like decline in the throughput curve. In contrast, the EB-LEACH algorithm, through the use of energy and distance weighting factors, effectively delays the failure of the first node (to 854 rounds), while simultaneously reducing reliance on any single node by optimizing the spatial distribution of cluster heads via an adaptive competition radius mechanism. Consequently, the throughput curve is smoother, with a much smaller sudden drop compared to LEACH and LEACH-C, demonstrating the algorithm’s advantages in load balancing and network robustness.

The experimental results show that the EB-LEACH algorithm outperforms other algorithms in terms of both data collection stability and sustainability. During the first 800 rounds of stable network operation, all three algorithms maintained a high data delivery rate (greater than 95%). Data throughput dropped sharply by the 800th round of the simulation, and, by the 1200th round, the packet delivery success rate had fallen to only 42.5%. The LEACH-C algorithm began to show a noticeable decline after the 1000th round.

The EB-LEACH algorithm still achieved a data delivery rate of over 78% after 1500 rounds, and the number of data packets transmitted throughout its entire life cycle increased by 118.3% and 52.7% compared to LEACH and LEACH-C, respectively. This advantage is primarily due to the stable cluster structure formed by the dynamic cluster head competition mechanism in the EB-LEACH algorithm, which effectively prevents transmission failures caused by the uneven distribution of cluster heads.

Based on the experimental results described above, the EB-LEACH algorithm demonstrates significant advantages across three key performance metrics: network lifetime, energy efficiency, and data throughput. Table 2 provides a quantitative comparison of the performance of the three algorithms.

As shown in Table 2, LEACH-C, as a typical centralized clustering routing protocol, relies entirely on the aggregation node to select cluster heads and partition the network. At the beginning of each round, all sensor nodes upload their remaining energy and location information to the base station; the base station then calculates the network-wide average energy and removes nodes with energy levels below the average. Subsequently, a simulated annealing algorithm is used to optimize the distribution of candidate cluster heads, minimizing the total communication distance within each cluster and finally broadcasting the clustering results to the entire network. Compared to the traditional LEACH algorithm, LEACH-C achieves a more uniform distribution of cluster heads, though it still has inherent limitations: the algorithm uses a fixed screening threshold throughout the network operation cycle and lacks adaptive screening criteria; furthermore, all nodes must upload status information in every round, resulting in significant control overhead. Additionally, LEACH-C cannot dynamically adjust the coverage radius based on node location, leading to excessive energy consumption by edge nodes. These inherent shortcomings make LEACH-C poorly suited for long-term monitoring scenarios in agricultural fields. The superior performance of the EB-LEACH algorithm is evident in Table 2. The innovative design philosophy of this algorithm involves protecting low-energy nodes through an energy factor, optimizing network topology via a distance factor, and balancing network load through a dynamic competition mechanism. The combined effect of these mechanisms enables the algorithm to effectively adapt to the practical requirements of beet field monitoring networks, thereby providing reliable communication support for Beidou- and ZigBee-based beet environmental monitoring systems.

### 4.3. Program Design for the Data Acquisition Node

**Figure 15:**
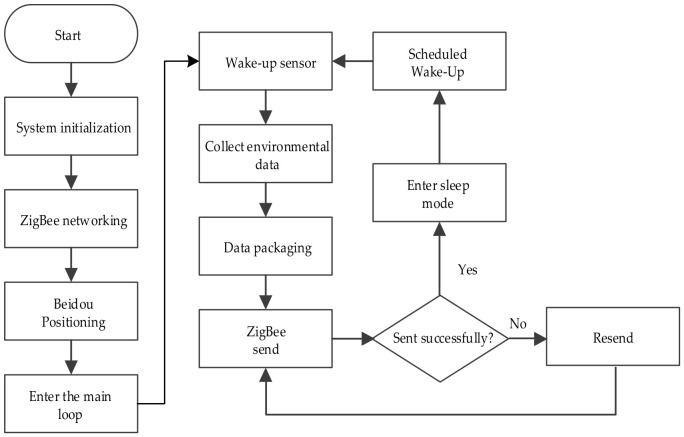
Flowchart of the main program for the data acquisition node.

The data acquisition node program runs on an STM32 microcontroller (STMicroelectronics, Geneva, Switzerland) using bare-metal programming. It primarily consists of several key states, including system initialization, network connection, data acquisition, data transmission, and sleep/wake-up. After power-up, the node first initializes basic peripherals such as the system clock, GPIO, UART, I2C, and ADC, followed by the sequential initialization of each sensor module and the ZigBee communication module. Once initialization is complete, the node searches for and joins the ZigBee network to obtain its network address. It then enters the main loop, executing the “wake-up–acquisition–transmission–sleep” workflow at a preset interval, as illustrated in Figure 15.

### 4.4. Gateway Aggregation Node Software Design

**Figure 16:**
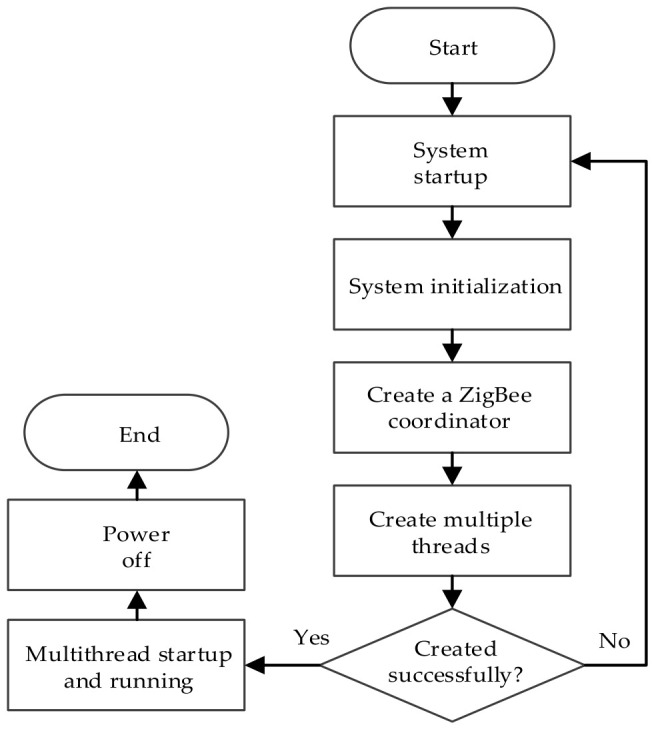
Flowchart of the gateway aggregation node main program.

The gateway aggregation node runs on the embedded Linux system of the IMX6ULL Pro core board and is responsible for functions such as ZigBee network management, data aggregation and processing, Beidou remote transmission, and command forwarding. The entire software system employs a multithreaded architecture, with each module operating in parallel to collaboratively perform data aggregation and remote transmission tasks. The overall process is illustrated in Figure 16.

## 5. System Results Testing and Analysis

**Figure 17:**
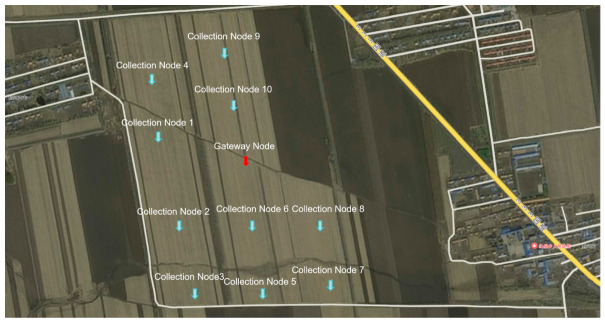
Node deployment diagram.

System testing was conducted at a sugar beet experimental field in Hongxing Township, focusing primarily on the analysis of four key performance indicators: communication range, data packet loss rate, power consumption, and measurement error. The measurement area measured 3.1 km in length by 2.5 km in width, with a deployment configuration as shown in Figure 17. The nodes adopt a mesh topology, comprising 10 data collection nodes and one gateway node. The data collection nodes were configured to collect data every 5 min. The ZigBee modules operated on the 2.4 GHz global ISM band, with a transmission power set to 5 dBm. The ideal line-of-sight communication range reached 500 m, with monitoring data transmitted every 20 min. The distance between the gateway node and the farthest data collection node (No. 7) was 2.3 km. 

### 5.1. System Communication Range Test

To evaluate the communication performance of the ZigBee module under different environmental conditions, communication range tests were conducted in both unobstructed and obstructed environments. During the tests, the gateway node was kept stationary while the data collection node was moved away from it, starting at a distance of 100 m and then gradually increasing. Signal strength and communication success rates were recorded at each distance, with 100 tests performed at each distance point. The test results are shown in Table 3.

Our test results indicate that, as the communication distance increases, both signal strength and communication success rates decline in both unobstructed and obstructed environments. Communication performance remains relatively stable in unobstructed environments, with a success rate of over 90% within 500 m. In obstructed environments, however, signal attenuation occurs more rapidly due to the obstruction caused by vegetation, resulting in a more significant drop in communication success rates. When deploying this system, keeping the distance between nodes within 300 m ensures a communication success rate of over 95%.

### 5.2. System Packet Loss Rate Test

**Figure 18:**
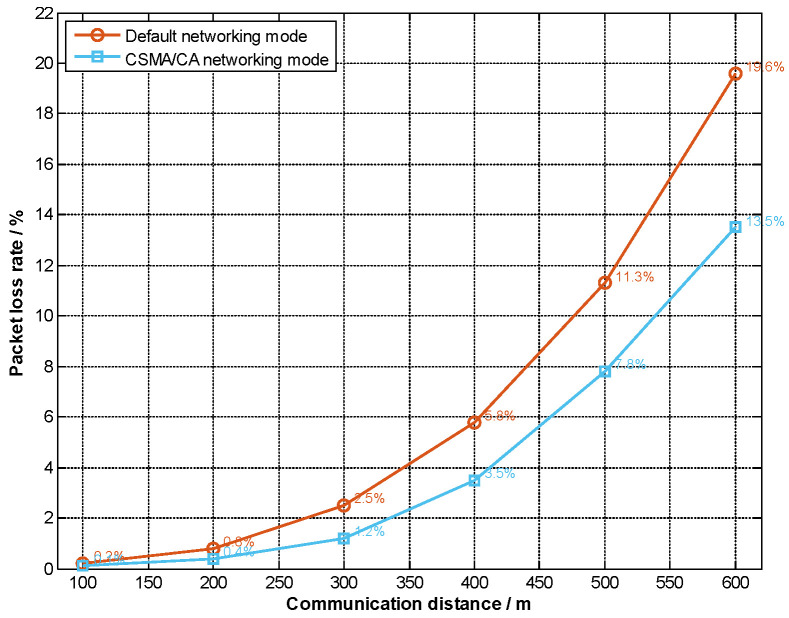
ZigBee node packet loss rate test results.

Packet loss rate is a key metric for evaluating the data transmission performance of wireless sensor networks. To test the actual performance of the ZigBee system in a sugar beet field environment, comparative experiments were conducted using both the default ZigBee networking mode and a CSMA/CA-based networking mode. The test results are shown in Figure 18.

With the gateway node fixed in position, nodes were deployed at varying distances. Each node transmitted data to the gateway at a frequency of one packet every 10 s, sending a total of 1000 packets. The gateway received the data in real time and recorded packet loss.

Our test results show that, as the communication distance increases, the packet loss rates for both networking modes gradually rise. However, the CSMA/CA network consistently exhibits a lower packet loss rate across all distances. At a distance of 300 m, the packet loss rate for the default network was 2.5%, while that for the CSMA/CA network was only 1.2%, representing a performance improvement of approximately 52%. At a distance of 500 m, the packet loss rate for the CSMA/CA network was 7.8%, a 31% reduction compared to the default network’s 11.3%, validating the effectiveness of the CSMA/CA mechanism in reducing channel collisions and improving transmission reliability.

### 5.3. System Power Consumption Testing

The sugar beet environmental monitoring system requires data collection nodes to operate stably in the field over the long term. The nodes’ power consumption primarily consists of three components: sensor data acquisition, node standby/sleep mode, and ZigBee data transmission. To test the nodes’ actual operating power consumption, 10 nodes were deployed in a sugar beet field for a 30-day field power consumption test. The data collection nodes are powered by 10,000 mAh lithium batteries, while the software design reduces the overall power consumption by adjusting sensor sampling frequencies and data transmission intervals. Given that environmental changes in beet fields occur relatively slowly, the data collection nodes were configured to collect data every 5 min, with the ZigBee module transmitting monitoring data every 20 min, resulting in four data collections per operational cycle. The power consumption parameters of the system’s data acquisition nodes over a 20-min period are shown in Table 4.

After 30 days (approximately 2160 cycles) of continuous system operation, the remaining charge of the lithium battery was measured at approximately 6800 mAh, meaning that, over the 30-day period, the battery consumed approximately 3200 mAh of charge, accounting for 32% of its total capacity. The system can operate continuously for approximately 90–100 days, meeting the monitoring requirements for a full growth cycle of sugar beets.

### 5.4. Sensor Error Testing

The error measurement for this system was conducted in two phases: first, system error testing was performed in the laboratory, followed by field testing after deploying the system in the sugar beet experimental fields of Hongxing Township. To ensure the accuracy of the collected data, all sensors underwent laboratory calibration prior to use.

Temperature and humidity sensors were calibrated with a high-precision constant temperature and humidity chamber (Model HWS-600, Hangzhou Chuanyi Instrument Co., Ltd., Hangzhou, China); light sensors were calibrated at multiple points using a metrological-grade standard illuminance meter (Model TES-1330A, TES Electrical Electronic Corp., Taipei, China); dissolved oxygen sensors were calibrated at zero and full scale using the saturated brine method (Model JPB-607A, Shanghai Leici Instrument Co., Ltd., Shanghai, China); and barometric pressure sensors underwent digital offset compensation using a standard barometer (Model DYM3, Shanghai Meteorological Instrument Factory, Shanghai, China). In addition to laboratory calibration, recalibration was performed during on-site deployment. This process effectively corrected systematic errors, reasonably suppressed random errors, and minimized the interference of gross errors, thereby improving the measurement accuracy of sensors.

#### 5.4.1. Sensor Laboratory Error Testing

In the laboratory, calibrated sensors were used to measure five parameters—temperature, humidity, light intensity, atmospheric pressure, and dissolved oxygen—under various environmental conditions. Thirty sets of data were continuously recorded for each parameter, with every 10 sets constituting a statistical unit. The mean absolute error and maximum measurement error were then calculated. The test results and analysis for the temperature and humidity sensors are shown in Table 5. In Table 5 the average relative measurement error for temperature across the full range does not exceed 1.61%, and the maximum relative measurement error does not exceed 2.65%. The average relative measurement error for relative humidity across the full measurement range does not exceed 2.30%, and the maximum relative measurement error does not exceed 3.53%.

These values are significantly lower than the acceptance criteria of ≤5.00% for temperature and ≤10.00% for humidity specified in agricultural industry standards. Table 6 presents the error analysis for light intensity measurements.

Table 7 presents the error analysis for air pressure measurements, and Table 8 presents the error analysis for dissolved oxygen measurements, as shown below.

Based on the error test analysis, the average relative measurement error for light intensity measurements in Table 6 does not exceed 4.1%, and the average maximum relative measurement error is controlled at 6.53%. In Table 7, the average relative measurement error for air pressure does not exceed 0.06%, and the maximum relative measurement error does not exceed 0.12%, which is well below the 1.00% acceptance criterion specified in meteorological industry standards. In Table 8, the measurement error for dissolved oxygen is less than 7.14%. All errors fall within the sensor’s nominal accuracy range, meeting the application requirements for sugar beet environmental monitoring.

#### 5.4.2. Error Testing After Sensor Deployment

**Figure 19:**
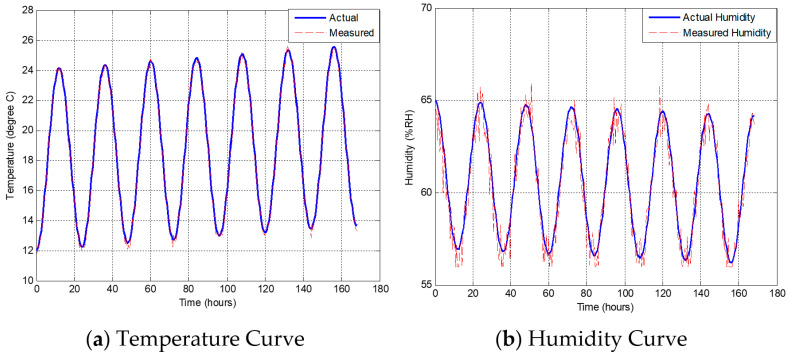
Comparison chart of temperature and humidity measurements.

To further analyze the operational stability of the system’s sensors, they were deployed in a sugar beet experimental field in Hongxing Township for a 7-day data collection period totaling 168 h, with data collected every 5 min. The laboratory-grade standard calibration instruments mentioned above were carried to the experimental field. The readings of these instruments were taken as standard values, and then the measured data were compared with the standard values. The results of the temperature and humidity measurements are shown in Figure 19, where (a) displays a comparison graph of node temperature measurements.

**Figure 20:**
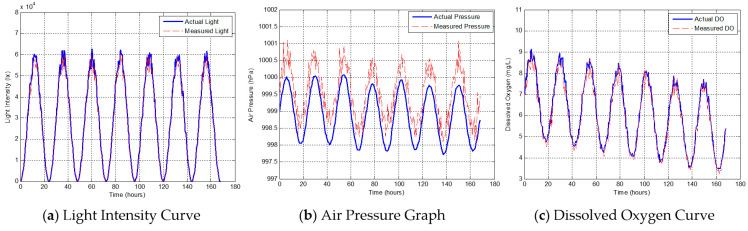
Comparison of light intensity, atmospheric pressure, and dissolved oxygen.

The trend in temperature readings closely matches the actual temperature values, with the two curves essentially overlapping. As the duration of data collection increases, the amplitude of fluctuations remains largely consistent, and the maximum relative measurement error consistently stays within 3%. This indicates that the monitoring system exhibits high accuracy and stability in temperature monitoring and can accurately reflect the diurnal variation patterns of field temperatures. The trend in humidity readings recorded in Figure 19b closely matches the trend in actual humidity values, with the maximum relative measurement error for humidity within 4.2%. The system can precisely capture the pattern where humidity decreases as temperature rises, meeting the accuracy requirements for humidity measurement in sugar beet field environmental monitoring. However, the measurement errors in outdoor conditions are significantly larger compared to those in laboratory conditions. This is because the laboratory environment is stable and free from external interference, with measurement errors caused only by the sensor’s inherent systematic bias and random noise, resulting in a higher overall accuracy. Therefore, the maximum relative errors in the field are significantly greater than those in the laboratory measurements. The results of comparing the field measurements of light intensity, air pressure, and dissolved oxygen in the system with standard values are shown in Figure 20.

In Figure 20, the standard value curve is used as a reference benchmark to compare and analyze the actual measurement deviations of the sensor under real-world conditions. In the illuminance measurement comparison curve shown in Figure 20a, the maximum relative error is less than 6.8%, the standard value exhibits regular diurnal variations, and the curve is smooth and continuous. However, due to sensor system bias, interference from ambient stray light and uneven dust adhesion, as well as inconsistent field-of-view aperture, mismatched spectral response, slight installation attitude difference, and inherent circuit systematic error between the self-developed sensor and the reference device, obvious deviations occur in light-intensity measurement, the actual measurement data exhibit significant fluctuations and generally fall below the standard true value. In Figure 20b, the baseline values for air pressure are relatively high. Compared to parameters such as temperature and light intensity, even small absolute errors can result in significant fluctuations in the graph. However, based on the error data, the maximum relative error for this air pressure measurement is less than 0.13%, making it the most accurate among all sensors. In Figure 20c, the dissolved oxygen sensor shows a continuous and gentle variation in the standard curve. This can be explained by the fact that in April, as the temperature continues to rise in the experimental field, the deep-frozen soil thaws continuously, and the recharge of soil groundwater gradually increases. The dissolved oxygen measured in this study is the oxygen content dissolved in soil pore water. At a soil depth of about 30 cm, the newly produced soil water from thawing frozen soil continuously transports exogenous dissolved oxygen. Its replenishment effect outweighs the negative impact of the decrease in saturated dissolved oxygen capacity of water caused by rising temperature. Meanwhile, the oxygen consumption of soil microorganisms and sugar beet root respiration is relatively weak in early spring. For this reason, soil dissolved oxygen increases synchronously with the rise in temperature at this stage. As sugar beet roots gradually develop and thicken, the oxygen consumption for plant growth continues to rise, which leads to a gradual decline in soil dissolved oxygen content. After the complete conclusion of the freeze–thaw period, the soil environment becomes stable, and the relationship between dissolved oxygen and temperature conforms to the conventional physical rule: the higher the water temperature, the lower the saturated dissolved oxygen content in water, showing a negative correlation between the two. In laboratory-controlled conditions, the soil is homogeneous with constant temperature and humidity, and the burial state of the two probes can be strictly consistent, leading to minor measurement deviation. However, in the complex environment of the experimental field, inconsistent soil contact compactness, micro-soil heterogeneity, different response speeds and temperature compensation characteristics between the two probes are significantly amplified by irrigation, weather fluctuation and diurnal temperature variation, which eventually results in much larger deviations than those in the laboratory environment, the maximum relative error does not exceed 7.50%, though the actual measurement curve exhibits significant fluctuations.

These results indicate that, although certain deviations exist during the measurement process, the error ranges of all sensors remain within reasonable fluctuation limits. The overall trend of the measurement curves is highly consistent with the standard true values, and data analysis and calculations confirm that the maximum relative errors meet the requirements of their respective fields. The sensors can accurately reflect the dynamic patterns of environmental parameters and demonstrate good reliability and practicality. In the future, noise can be further suppressed and measurement deviations reduced through filtering algorithms and hardware protection measures, thereby enhancing monitoring stability.

## 6. Conclusions

In this paper, we designed a beet environmental monitoring system based on Beidou and ZigBee and proposed an efficient ZigBee routing algorithm called EB-LEACH. Through simulation and experimentation, we demonstrated that the EB-LEACH algorithm offers significant advantages over traditional LEACH in terms of network lifetime, energy efficiency, and data throughput. Finally, through integrated hardware and software design, a fully functional and reliable sugar beet environmental monitoring system was constructed. Our test results indicated that the system is fully functional and reliable, capable of meeting the practical requirements for environmental monitoring in sugar beet fields.

## Figures and Tables

**Table 1:** Simulation parameter settings.

Category	Parameter Name	Parameter Value	Explanation
Network Topology	Simulation Area	100 × 100 m^2^	Square monitoring area
	Number of Nodes	100	Random uniform distribution
	Convergence Nodes	(50, 50)	Located at the center of the area
Energy Parameters	Initial Energy	2 J	All nodes are identical
	Packet Size	512 bytes	Fixed-length packets
	Control Packet Size	32 bytes	Cluster head election messages
Algorithm Parameters	Cluster Head Ratio	0.05	5% of nodes serve as cluster heads per round
	Energy Weight α	0.6	Energy factor weight
	Distance Weight β	0.4	Distance factor weight
Simulation Settings	Total Iterations	2000	Maximum number of simulation rounds
	Number of Repeats	10	Results are averaged

**Table 2:** Comprehensive comparison of algorithm performance.

Performance Metrics	LEACH	LEACH-C	EB-LEACH	Percentage Increase Compared to LEACH (%)
Number of rounds until the first node fails	456	612	854	87.3%
Network half-life rounds	525	780	1105	110.48%
Number of rounds until the entire network fails	1524	1768	1986	30.3%
Remaining energy rate of a thousand rounds	5.32%	11.13%	21.64%	16.32%
Total packet delivery volume (×10^3^)	86.5	122.3	188.8	118.3%

**Table 3:** ZigBee node communication performance test.

Test Environment	Unobstructed Environment		Environments with Obstructions	
Communication Range/m	RSSI/dBm	Communication Success Rate/%	RSSI/dBm	Communication Success Rate/%
100	−51	98.6	−58	98
200	−61	98.3	−69	96
300	−73	98	−81	92
400	−82	95	−93	87
500	−91	91	−102	79
600	−98	84	−108	68

**Table 4:** Power consumption parameters of data collection nodes.

Work Status	Average Current/mA	Duration/s	Energy Consumption Within 20 min/mAh
Data collection (4 times)	45	30	0.375
ZigBee send	125	12	0.417
Low-power sleep mode	0.055	1158	0.018
MCU & peripherals standby	2	1200	0.667
Total for the period		1200	1.477

**Table 5:** Analysis of measurement errors in air temperature and humidity.

Parameter	Reference Value	Measured Average	Mean Absolute Error	Maximum Measurement Error	Average Relative Measurement Error	Maximum Relative Measurement Error
Temperature/°C	15.1	14.9	±0.2	±0.4	1.32%	2.65%
Temperature/°C	19.8	20.1	±0.3	±0.5	1.52%	2.53%
Temperature/°C	24.9	24.5	±0.4	±0.6	1.61%	2.41%
Humidity/%RH	56.3	57.1	±0.8	±1.5	1.42%	2.66%
Humidity/%RH	59.8	58.7	±1.1	±1.9	1.84%	3.18%
Humidity/%RH	65.2	63.7	±1.5	±2.3	2.30%	3.53%

**Table 6:** Analysis of measurement errors in light intensity.

Reference Value/*lx*	Measured Average/*lx*	Mean Absolute Error/*lx*	Maximum Measurement Error/*lx*	Average Relative Measurement Error	Maximum Relative Measurement Error
90,000	86,940	±3060	±5850	3.40%	6.50%
12,500	11,988	±513	±975	4.10%	7.80%
30,000	29,100	±900	±1590	3.00%	5.30%

**Table 7:** Analysis of air pressure measurement errors.

Reference Value/hPa	Measured Average/hPa	Mean Absolute Error/hPa	Maximum Measurement Error/hPa	Average Relative Measurement Error	Maximum Relative Measurement Error
999.4	1000	±0.6	±1.2	0.06%	0.12%
999	999.6	±0.6	±1.1	0.06%	0.11%
998	998.5	±0.5	±1.0	0.05%	0.10%

**Table 8:** Analysis of measurement errors in dissolved oxygen.

Reference Value (mg/L)	Measured Average (mg/L)	Mean Absolute Error (mg/L)	Maximum Measurement Error (mg/L)	Average Relative Measurement Error	Maximum Relative Measurement Error
10	9.85	±0.3	±0.5	3.00%	5.00%
6.5	6.35	±0.2	±0.35	3.08%	5.38%
3.5	3.35	±0.15	±0.25	4.29%	7.14%

## Data Availability

The raw data supporting the conclusions of this article will be made available by the authors on request.
